# Construction Notes

**Published:** 1896-01-11

**Authors:** 


### CONSTRUCTION NOTES.

### New Cottage Hospital at Farnham.

This cottage hospital has just been opened, having been built by funds provided under the bequest of the late Mr. George Trimmer. The building commands a southern aspect, and the wards, male and female, open on to a verandah. The accommodation in each case is for three beds. The wards are 21 ft. by 14 ft. On the ground floor are also a small operating-room and an accident ward. A convalescents' room is situated upstairs. The total cost of the building was £2,000.

